# Genome of *Cnaphalocrocis medinalis* Granulovirus, the First Crambidae-Infecting Betabaculovirus Isolated from Rice Leaffolder to Sequenced

**DOI:** 10.1371/journal.pone.0147882

**Published:** 2016-02-05

**Authors:** Guangjie Han, Jian Xu, Qin Liu, Chuanming Li, Hongxing Xu, Zhongxian Lu

**Affiliations:** 1 Department of Biological Pesticides, Jiangsu Lixiahe Institute of Agricultural Sciences, Yangzhou, 225007, PR China; 2 State Key Laboratory Breeding Base for Zhejiang Sustainable Pest and Disease Control, Institute of Plant Protection and Microbiology, Zhejiang Academy of Agricultural Science, Hangzhou, 310021, PR China; University of Alabama at Birmingham, UNITED STATES

## Abstract

*Cnaphalocrocis medinalis* is a major pest of rice in South and South-East Asia. Insecticides are the major means farmers use for management. A naturally occurring baculovirus, *C*. *medinalis* granulovirus (CnmeGV), has been isolated from the larvae and this has the potential for use as microbial agent. Here, we described the complete genome sequence of CnmeGV and compared it to other baculovirus genomes. The genome of CnmeGV is 112,060 base pairs in length, has a G+C content of 35.2%. It contains 133 putative open reading frames (ORFs) of at least 150 nucleotides. A hundred and one (101) of these ORFs are homologous to other baculovirus genes including 37 baculovirus core genes. Thirty-two (32) ORFs are unique to CnmeGV with no homologues detected in the GeneBank and 53 tandem repeats (TRs) with sequence length from 25 to 551 nt intersperse throughout the genome of CnmeGV. Six (6) homologous regions (*hrs*) were identified interspersed throughout the genome. *Hr2* contains 11 imperfect palindromes and a high content of AT sequence (about 73%). The unique ORF28 contains a coiled-coil region and a zinc finger-like domain of 4–50 residues specialized by two C2C2 zinc finger motifs that putatively bound two atoms of zinc. ORF21 encoding a chit-1 protein suggesting a horizontal gene transfer from alphabaculovirus. The putative protein presents two carbohydrate-binding module family 14 (CBM_14) domains rather than other homologues detected from betabaculovirus that only contains one chit-binding region. Gene synteny maps showed the colinearity of sequenced betabaculovirus. Phylogenetic analysis indicated that CnmeGV grouped in the betabaculovirus, with a close relation to AdorGV. The cladogram obtained in this work grouped the 17 complete GV genomes in one monophyletic clade. CnmeGV represents a new crambidae host-isolated virus species from the genus *Betabaculovirus* and is most closely relative of AdorGV. The analyses and information derived from this study will provide a better understanding of the pathological symptoms caused by this virus and its potential use as a microbial pesticide.

## Introduction

The rice leaffolder, *Cnaphalocrocis medinalis*Güenée (Lepidoptera: Crambidae), is a migratory and important insect pest of rice in Asia [1, 2]. The larvae fold the leaves, feed on the photosynthetic leaf tissues in the folded leaves and such damages can result in reduction of rice yields [3]. In China frequent outbreaks have occurred in rice production regions and have caused rice yield reduction and farmers’ overuse of insecticides. Insecticide control is the main measure farmers in China use and the pests have developed resistance to some insecticides [4]. The CnmeGV belonging to the family of *Baculoviridae*, was isolated from the infected caterpillars collected from fields in China recently. Bioassay showed that CnmeGV is a highly virulent baculovirus and suggested the potential of its use as an environmentally friendlier microbial agent for future rice leaffolder management [5].

*Baculoviridae* is a family ofrod-shaped baculoviurs with circular, covalently closed double-stranded DNA genomes, which has been successfully applied for the control of some agricultural and forest insect pests [6]. Based on phylogeny and host specificities, Baculoviridae is divided into four genera: *Alphabaculovirus* (lepidopteran-specific nucleopolyhedrovirus, NPVs), *Betabaculovirus* (lepidopteran-specific granulovirus, GVs), *Gammabaculovirus* (hymenopteran-specific NPVs) and *Deltabaculovirus* (dipteran-specific NPV) [7]. *Alphabaculovirus*is further subdivided into groups I and II according to the phylogenetic analysis of the *lef-8*, *lef-9* and *polh/gran* genes [8]. *Betabaculovirus*is classified into three types based on the tissue tropism [9]. To date, the complete genomes of more than 51 NPVs and 17 GVs are published or available in GenBank.

GVs are more specific than NPVs, which have been reported only from Lepidoptera [10]. Partly because of the difficulty of establishing cell lines that are permissive for GV infection, the molecular biology and genetics of GVs have been less well studied than those of NPVs [11]. CnmeGV is a new isolate and is an effective baculovirus pathogen but less studied and genomic information is lacking. In this paper we present the complete sequence and morphological characterization of the CnmeGV genome and compared them to other baculoviruses using genomic and phylogenetic analyses. This is the first completely sequenced betabaculovirus isolated from a crambidae host to be reported.

## Results and Discussion

### Sequence analysis of the CnmeGV genome

**Fig 1:**
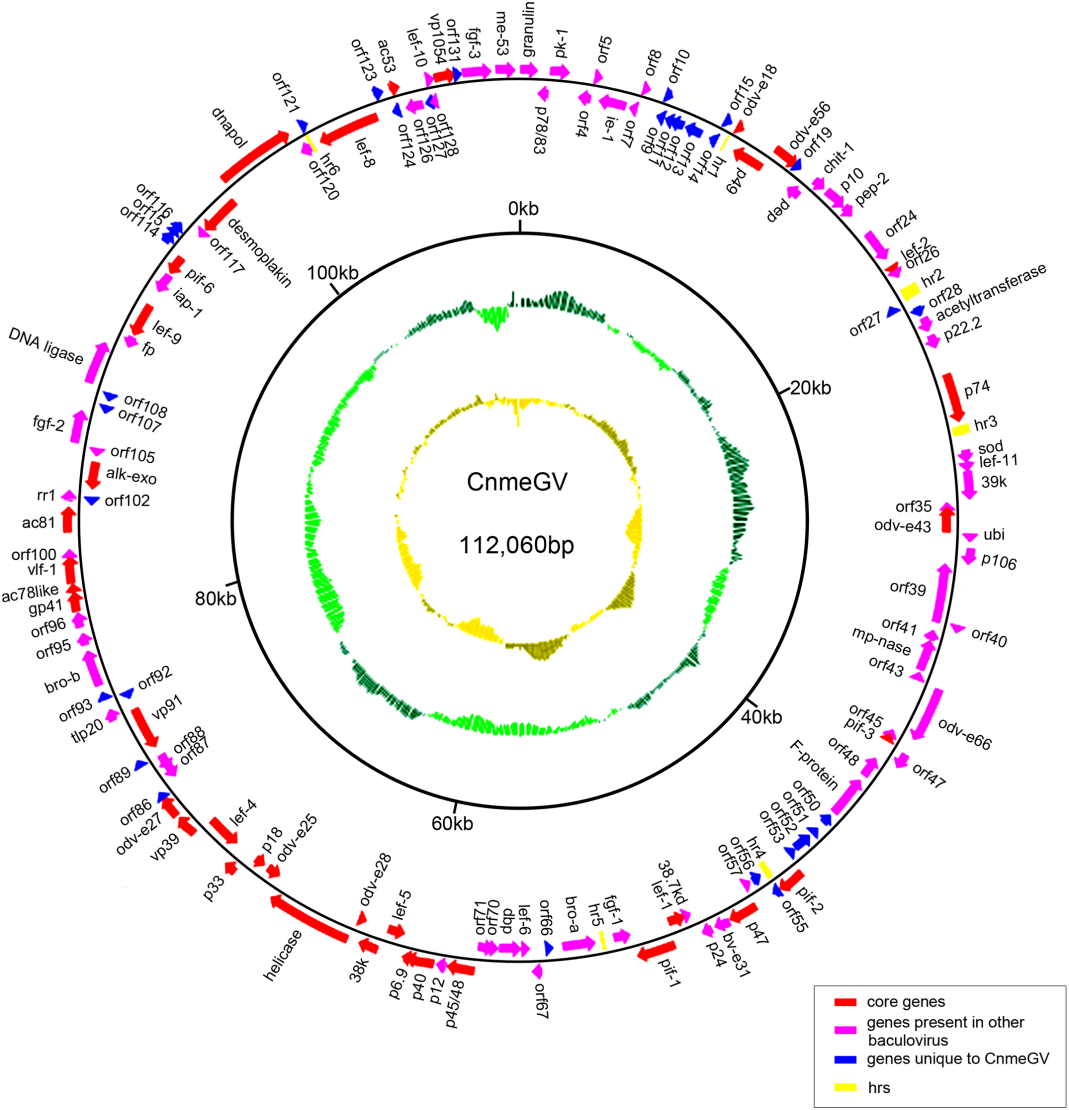
Circular map of the CnmeGV genome. ORFs and transcription direction are indicated as arrows. Core genes were indicated by red arrows, genes present in other baculovirus were indicated by pink arrows, unique genes were indicated by blue arrows and *hrs* were indicated by yellow squares. The innermost circle shows GC skew, which indicates possible locations of the DNA leading strand, lagging strand, replication origin, and replication terminal during DNA replication. Below average GC skew is light orange and above average dark orange. The next innermost circle is a GC plot, with light green representing below average GC content, and dark green indicating above average GC content.

A total of 53,359 reads from post-filter sequencing libraries were used for genome assembly by the hierarchical genome-assembly process (HGAP). The genome of CnmeGV was sequenced and was registered as the first complete sequence of a crambidae infecting betabaculovirus in GenBank (Accession number KP658210). The genome consisted of 112,060 bp, which was within the sizes of the 17 sequenced betabaculovirus genomes ranging from 99,657 bp in AdorGV [12] to 178,733 bp inXcGV [13] (Table 1). The G+C content of CnmeGV genome was 35.2%, close to the lowest one estimated for betabaculovirus members which ranged between 32.5% in CrleGV and 45.2% in CpGV. However, no correlation was found between these data and the biological properties. In the criteria for selecting ORFs there should be methionine-initiated ORFs of at least 50 codons having minimal overlap with other ORFs [14], 133 putative ORFs were identified and were numbered from the ATG start codon of the *granulin* gene in a clockwise direction (S1 Table). Coding sequences represented 85.1% of the genome of CnmeGV similar to CpGV [15]. Seventy (70) ORFs were in the same orientation as the *granulin* ORF and 63 were opposite, indicating that CnmeGV ORFs have no obvious preferred orientation. Helicase (ORF79) is the longest sequence gene encoding 1162 amino acids, while ORF8 is the shortest in CnmeGV genome. The circular map of the CnmeGV genome was established and shown in Fig 1.

**Table 1:** All species from the genus *Betabaculovirus* completely sequenced to date*.

Virus species	Host family	Size (bp)	ORFs	Accession	Tandem repeats number	Percentage of genome (%)	Authors
*Adoxophyes orana* granulovirus	Tortricidae	99,657	119	AF547984	22	2.31	Wormleaton et al.
*Agrotis segetum* granulovirus Xinjiang	Noctuidae	131,680	132	AY522332	14	0.76	Ai et al.
*Choristoneura occidentalis* granulovirus	Tortricidae	104,710	116	DQ333351	56	8.38	Escasa et al.
*Clostera anachoreta* granulovirus	Notodontidae	101,487	123	HQ116624	9	0.59	Liang et al.
*Clostera anastomosis L*. granulovirus	Notodontidae	101,818	123	KC179784	4	0.24	Liang et al.
*Cnaphalocrocis medinalis* granulovirus	Crambidae	112,060	133	KP658210	53	3.83	Han et al.
*Cryptophlebia leucotreta* granulovirus	Tortricidae	110,907	129	AY229987	46	4.66	Jehle et al.
*Cydia pomonella* granulovirus	Tortricidae	123,500	143	U53466	40	2.38	Crook et al.
*Epinotia aporema* granulovirus	Tortricidae	119,092	132	JN408834	22	1.14	Ferrelli et al.
*Erinnyis ello* granulovirus	Sphingidae	102,759	135	KJ406702	24	1.66	Ardisson-Araújo et al.
*Helicoverpa armigera* granulovirus	Noctuidae	169,794	179	EU255577	26	2.53	Harrison et al.
*Phthorimaea operculella* granulovirus	Gelechiidae	119,217	130	AF499596	134	7.65	Taha et al.
*Pieris rapae* granulovirus China	Pieridae	108,592	120	GQ884143	42	2.92	Wang et al.
*Plutella xylostella* granulovirus	Plutellidae	100,999	120	AF270937	32	9.38	Hashimoto et al.
*Pseudaletia unipuncta* granulovirus	Noctuidae	176,677	183	EU678671	20	1.40	Li et al.
*Spodoptera litura* granulovirus	Noctuidae	124,121	136	DQ288858	25	2.21	Wang et al.
*Xestia c-nigrum* granulovirus	Noctuidae	178,733	181	AF162221	27	1.82	Hayakawa et al.

*The same field of different types were not shown.

The putative proteins of those ORFs were predicted by BlastX search which had an E-value of less than 10^−6^ in NCBI. In total, 101 of the 133 putative ORFs encoding similar proteins are found in other organisms, while 32 of these were shown to be unique. Core Genes were a set of factors strongly conserved in the *Baculoviridae* family for they provide the essentials roles needed to complete the virus cycle [16]. When compared to the ORFs encoding the 37 described core proteins for *Betabaculovirus* genus [17], the 37 core genes were found in CnmeGV genome, representing the essential functions for replication and transcription; cell cycle interaction and/or arrest with host proteins; packaging and assembly; viral release; and oral infectivity. Baculovirus repeated ORFs (*bro* genes) were striking features of many baculovirus genomes. Two repeated *bro* genes were identified in the CnmeGV genome (ORF65, 94) and were designated as *bro-a*, *bro-b* respectively based on their order in the genome. This highly repetitive and conserved family might have functioned as DNA binding proteins that influenced host DNA replication or transcription and improve the infection capability of virus [18, 19].

### Replication genes

The core genes of CnmeGV involved in DNA replication, *alk-exo* (ORF104), *dnapol* (ORF119), *lef-1* (ORF62), *lef-2* (ORF25), *helicase* (ORF79), were detected. Other replication genes that belonged to lepidoptera baculovirus conserved genes discovered in CnmeGV were *dbp* (ORF69), *ie-1* (ORF6) and *me-53* (ORF133). Similarly, the *lef-7* gene was not found in CnmeGV while present in most of NPVs and only 4 GVs (HearGV, XcGV, PsunGV, SpfrGV). In the EpapGV genome, a protein, epap36, was found to have a match with PsunGV *lef-7*, but revealed a lower e-value (E = 0.54) [20].

The non-conserved baculoviurs gene *rr1* (ORF103) was also identified in CnmeGV. This putative protein had about 133 aa present and had lower identity than that of PhopGV rr1 (E = 7e-07, 33% amino acid identity) and CpGV rr1 (E = 0.088, 26% amino acid identity). Proteins of rr1 present in most NPVs generally have higher identity [21, 22]. In other GVs genomes, genes of *rr1* encoded proteins usually have about 609–782 aa and higher identity with NPVs (among 25%-53%). So, ORF103 in the CnmeGV genome might be a truncated sequence of the *rr1* gene.

### Transcriptional genes

Transcriptional genes presented as core genes in the *Baculoviridae* family, includes *lef-4* (ORF83), *lef-5* (ORF76), *lef-8* (ORF122), *lef-9* (ORF111), *p47* (ORF58) and *vlf-1* (ORF99) were detected in the CnmeGV DNA. Other genes, *39k* (ORF34), *lef-6* (ORF68), *lef-11* (ORF33) and *pk-1* (ORF3), related to the transcription process in all lepidopteran baculovirus were also identified in CnmeGV genome. The codes of CnmeGV ORF33, which was named *lef-11*, similar to ClanGV ORF47 (64% amino acid identity), might be necessary for efficient transcription as a gene for viral late gene expression [23]. Another late transcription gene *lef-10*, presented in most alphabaculoviruses (except SujuNPV, ClbiNPV and OrleNPV [24]) and betabaculoviruses, was also detected in the CnmeGV genome (ORF129). ORF129 also showed a 61% (E = 3e-18) and 39% (E = 0.003) amino acid identity to PrGV and AdorGV, respectively. *Lef-10* might be possibly the components of multi-subunit RNA polymerase that might be involved in late and very late transcription [25].

### Structural genes

All the core genes associated with structure were found in CnmeGV genome. The *pif*s, including *pif-1* (ORF63), *pif-2* (ORF54), *pif-3* (ORF46), *pif-4*/*odv-28* (ORF78), *pif-5*/*odv-e56* (ORF18), *pif-6* (ORF113) were found. *Pif* genes encode an essential structural protein of the occlusion-derived virus envelope [26]. In the early stages of virus infection, *pif-1*, *pif-2* and *pif-3* perform an essential function in association with *p74* [27]. In addition, 7 other conserved genes related to structure were also found in the CnmeGV genome: *fp* (ORF110), *p12* (ORF73), *p24* (ORF60), *tlp20* (ORF71), *F-protein* (ORF49), *granulin* (ORF1), and *p10* (ORF22). ORF1 was identified to reveal 86% amino acid identity to AnbiGV *granulin*, the major component of occlusion bodies as conserved baculovirus structural protein [28]. ORF22 in CnmeGV genome revealed 52% homologous to p10 of AdorGV. It shared a high conserve region at N- terminal (3–102 aa) and C- terminal (180–271 aa). P10 protein formed fibrillar structures, advantageous to occlusion body morphogenesis that result in the disseminating of occlusion bodies (OBs) [29]. In addition, this protein includes various common structural and functional domains: a coiled-coil region followed by a proline-rich domain, a variable region and finally a basic region at the C-terminal, which is characterized from different baculoviruses [13]. ORF22 also showed similarity to the *calyx*/*pep*/*pp34* genes of GVs. Therefore, more studies should be done to understand the role of the *p10* homologues in CnmeGV.

### Unique ORFs

Thirty two (32) ORFs appeared to be unique to CnmeGV compared to the rest of the members of Baculoviridae (ORF9, 10, 11, 12, 13, 14, 15, 19, 27, 28, 50, 51, 52, 53, 55, 56, 66, 86, 89, 92, 93, 102, 107, 108, 114, 115, 116, 121, 123, 124, 127, 131). The predicated proteins were peptides with no significant similarities to any other sequences in GenBank. Among these unique ORFs in the CnmeGV genome, 14 ORFs were in the same orientation as the *granulin* ORF and 18 in the opposite. Three (3) ORFs (ORF15, 27 and 131) presented a late promoter motif (GATA), suggesting expression at a late stage of viral infection. Early promoter motifs, including CAKT and TATAWAW, were also detected at upstream of the start codon in other ORFs. ORF14 was the longest sequence of the unique ORFs encoded for a putative protein of 271 aa. It had no significant BlastP hits, and had early promoter elements upstream of the first ATG (TATAAAT). ORF55 encoded the shortest hypothetical protein of 52 aa in unique proteins. An early promoter motif (ATTTATA) was also found 57 nt upstream ORF55. The proteins encoded with others ORFs also showed no significant BlastP hits. It seemed apparent that the CnmeGV shared much more unique genes. Whether these are functional ORFs of CnmeGV would require further experimentation.

The SMART program detected 15 unique ORFs that contained at least one region which encoded a limited set of amino acids of special domains. Three (3) ORFs (ORF107, 116, 124) were found with trans-membrane helix regions by the TMHMM v2.0 program. Thirteen (13) ORFs (ORF13, 14, 28, 55, 56, 66, 86, 92, 107, 108, 114, 123, 131) were detected with low-complexity regions (LCRs) by the SMART program. ORF56 encoded three LCRs with the longest one containing 61 aa within 62–122 aa. ORF28 was found to be a coiled-coil region by the COILS program. The coiled-coil segments lie in areas that are possibly playing a functionally important role [30]. Interestingly, the predicated protein of ORF28 also contained a domain of zinc finger-like of 4–50 residues specialized by two C2C2 zinc finger motifs that putatively bound two atoms of zinc. The function of this domain was hypothesized to involve protein dimerization [31], or suggested as an ubiquitin ligase [32], or necessary for DNA binding and zinc-dependent repression [33]. In addition, zinc fingers are typical motifs distributed in DNA/RNA regulatory proteins whereas the coordination of heavy metals is often a characteristic of different metallothioneins in some cases [17]. These assumptions would need further experimentation.

### Tandem repeats (TRs) and Homologous regions (*hrs*)

Tandem repeats (TRs) are DNA repeat sequences of each repeat unit located right next to each other, reflecting their origin in local duplications. These ubiquitous, unstable elements were found to combine characteristics of genetic and epigenetic changes that might facilitate organismal evolvability [34]. In the genome of PhopGV, 134 TRs were detected in a frequency of 7.65% in the genome. It was the highest TRs composed in the genomes of all betabaculovirus to date. The least TRs were detected in the genome of CalGV, which has 4 TRs with a frequency of 0.24% in genome (Table 1). In a screening of the CnmeGV genome for repeated sequences with TRs Finder [35], 53 TRs were found with sequence lengths from 25 to 551 nt. Fifteen (15) TRs were located in the coding region, 22 TRs were in the non-coding region and 16 TRs were in both the regions. All the 53 TRs contained 3.83% of genome of CnmeGV. These TRs in baculovirus genomes enhance the transcription of early gene in promoters and act as mediators for rapid phenotypic changes in coding sequences [34, 36].

**Fig 2:**
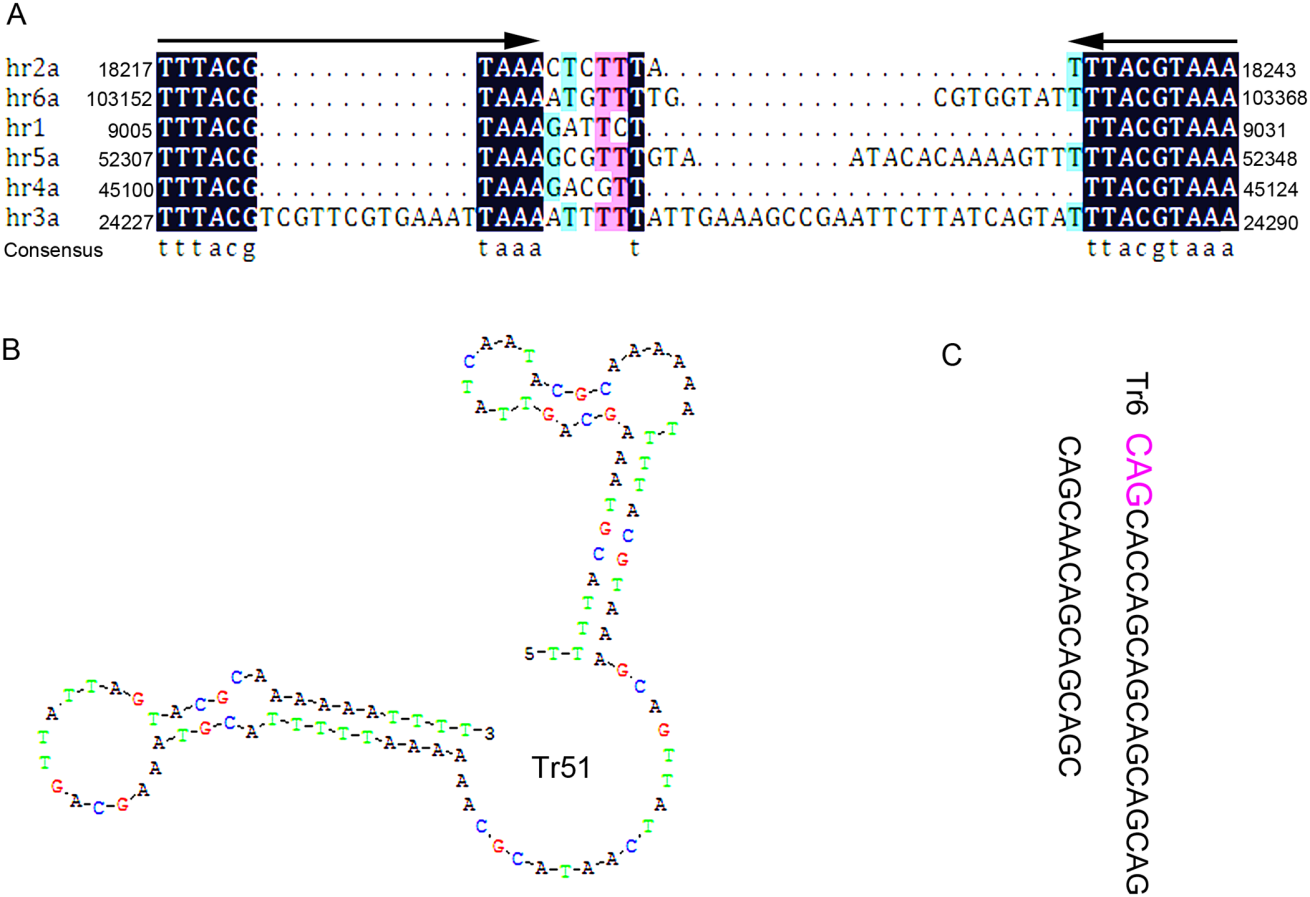
TRs and *hrs* analysis. **(A)** Alignment of the part of *hrs* in the CnmeGV genome. Palindromes within the repeats are indicated by arrows on the alignment. **(B)** Predicted secondary structure of the Tr51. **(C)** The repetitive sequence in coding regions of Tr6, CAG is repeating unit.

Mutations in these repeats often have fascinating phenotypic consequences [36]. The number of the repeating unit changes, recombination and replication slippage will bring about mutation in TRs [37]. *Tr51*, the least repeat unit of TRs in CnmeGV genome, contained 3 repeat units. The secondary structure of hairpin-loops was predicted by DNAMAN 8 (Minimum free energy of the structure is -14.01kcal/mol) (Fig 2B). This structure was a part of variability [36]. In addition to inherent instability, TR mutation can also be affected by external factors [38]. For example, CAG repeat stability is modulated by the chaperone protein hsp90 in the human cell. Hsp90 function can be overwhelmed by severe environmental stresses, resulting in a role of mediating an influence by the environment on TR mutation rates [39]. In the genome of CnmeGV, a CAG repeat unit in the TRs of *Tr6* contained 12.3 repeat units was found in the coding regions (Fig 2C). It coded a hypothetical protein of 333 amino acids. This might be possible that the correlation between CAG repeat units and the stability of the hypothetical protein would response to the environment. This assumption would need further verification.

In the baculovirus genomes sequenced so far, it is common to find 1 to 16 homologous regions (*hrs*) present. [40]. Generally, the *hrs* in baculoviruses are the intergenic repeats that play putative or demonstrated roles as enhancers of transcription and origins of replication [41]. Twenty seven (27) (within 6 *hrs*) imperfect palindromes were identified in CnmeGV genome. However, only 4 *hrs* were identified in ClanGV genome sequence [42]. The alignment of these sequences revealed a typical structure of palindrome. The alignment of these shorter palindromes shows that they have a 10 bp conserved inverted repeats (Fig 2A). Similarly, these palindrome structures of *hrs* were found in numerous GVs (EpapGV, CrleGV, AdorGV, and ChocGV) [43, 44, 15].

The largest intergenic region, which contains imperfect palindromes, was found between ORF26 and ORF27 in CnmeGV. It contained 757 bp in size and a high content of AT sequence (about 73%). Eleven (11) imperfect palindromes were identified in this region, which was assigned in *hr2*, but it revealed no significant homology in tBlastx searches.

### ORF21, with double chitin-binding domains

**Fig 3:**
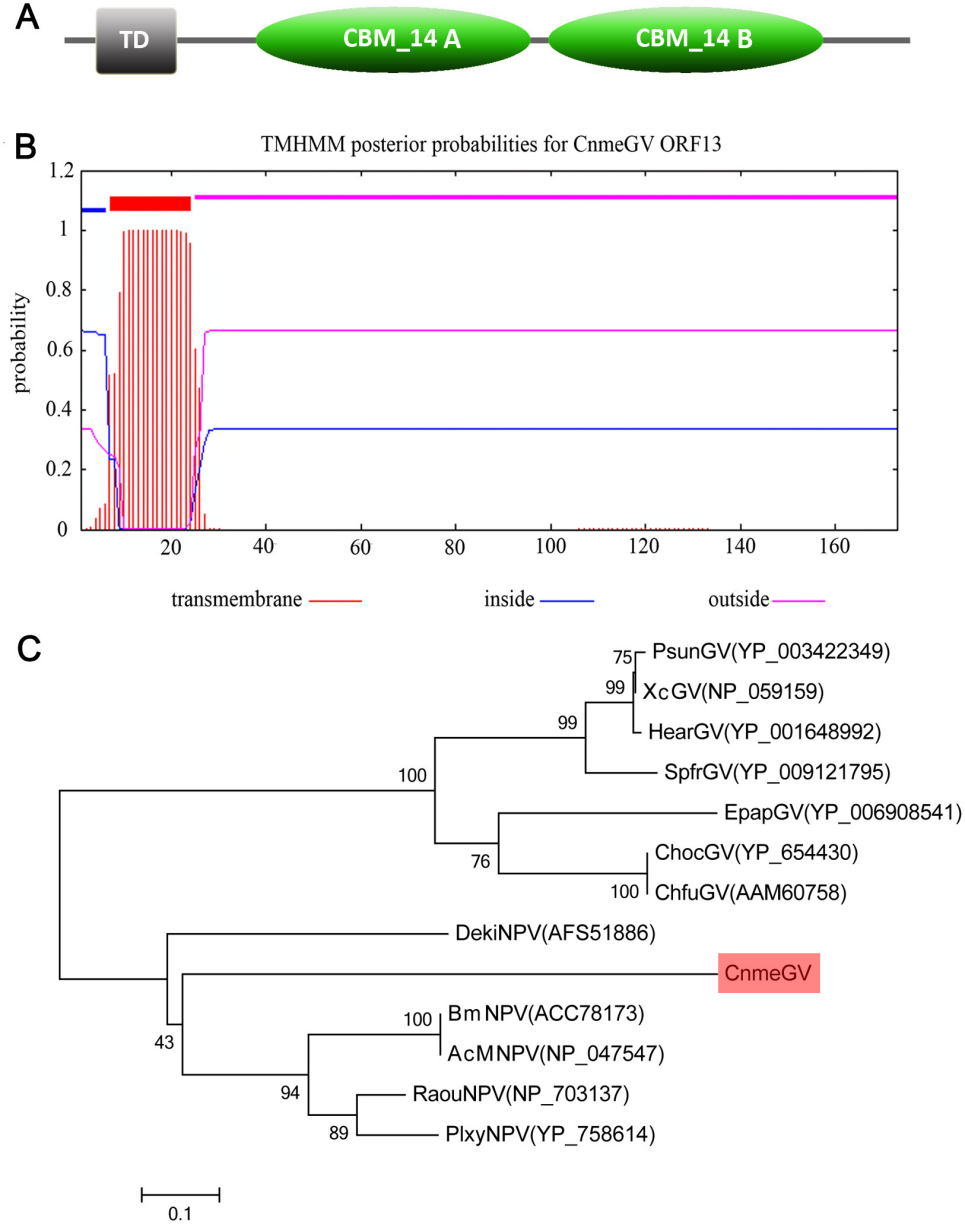
Structural domain and phylogenetic analysis of ORF21. **(A)** The structural organization of ORF21 protein. It contains a transmembrane helix domain (TD) and two carbohydrate-binding modules family 14 (CBM_14A and CBM_14B). **(B)** Transmembrane helix region was detected by the TMHAMM V2.0 program. **(C)**The NJ tree was inferred using the conserved amino-terminal region alignment of ORF21 gene for 13 baculoviruses. The postulated horizontal gene transfer (HGT) events are highlighted for CnmeGV.

Chitin is an important component of the insect cuticle and the peritrophic matrix (PM) lining the gut epithelium. The *chitinase* gene of baculovirus is usually expressed in the late phase of virus replication in insects that can hydrolyze chitin in the body of the insect that promotes terminal host liquefaction [45]. The CnmeGV ORF21 encoding a predicted protein of 173 aa with a size of 19.85 kDa is homologous to *chit-1* gene. A baculovirus consensus late promoter motif TTAAG was found at 8 nt upstream of the start codon ATG, indicating that ORF21 may express in the later stages of the infection cycle. ORF21 protein contains a trans-membrane helix region as detected by the TMHAMM V2.0 program (Fig 3B). The region started at position 7 aa and ended at position 24 aa. Moreover, two special domains, CBM_14A and CBM_14B, belonging to the carbohydrate-binding module family 14 (CBM_14) were found by the SMART to be located at the sites of 40–95 aa and 99–154 aa of the protein (Fig 3A).

**Fig 4:**
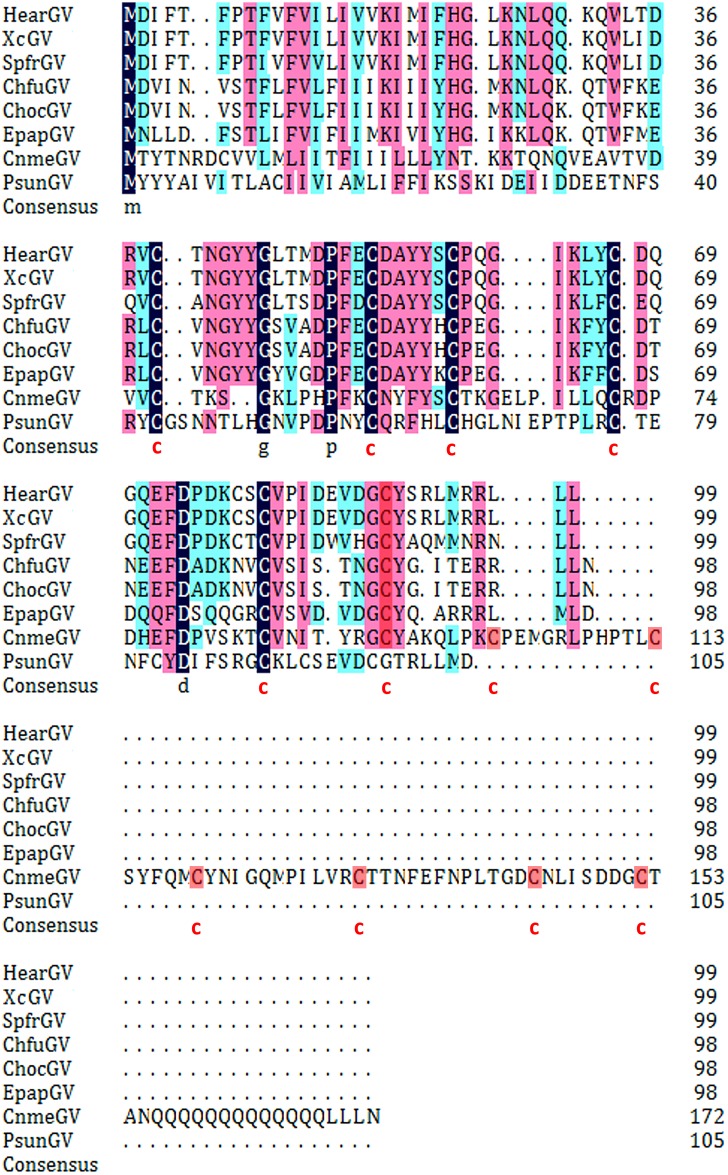
Multiple sequence alignment of proteins with the chit-binding domain. This domain contains one or two six-cysteine-containing motif that are indicated with red font. The alignment was generated by using ClustalX and edited using DNAMAN software. The sequence used were: HearGV gp010 (YP_001648992); XcGV ORF11 (NP_059159); SpfrGVchit-1 (YP_009121795); ChfuGV ORF9 (AAM60758); ChocGV gp009 (YP_654430); EpapGV chit binding protein (YP_006908541); PsunGV gp010 (YP_003422349).

The family of CBM_14 was known as the peritrophin-A domain found in chitin binding proteins, particularly, the PM proteins of insects and animal chitinases [46–48]. Homologous genes were also found in some other betabaculoviruses, but these genes only contained one chit-binding region (Fig 4). All the chitin-binding domains were characterized by processing a six-cysteine-containing motif: C-x(13,20)-C-x(5,6)-C-x(9,19)-C-x(10,14)-C-x(4,14)-C [49]. Comparing the homologous genes among sequenced betabaculoviruses using the BlastP and subsequently aligning by the ClustalX program, we found high similarity shared among HearGV, XcGV, SpfrGV, ChfuGV, ChocGV, EpapGV, except CnmeGV and PsunGV (Fig 4). Chitin-binding proteins encoded by baculoviruses might be involved in the virus-host interactions during the infection cycle [50]. The protein GP37 binding to the chitin of *Spodoptera litura* PM was found to facilitate virus infection by targeting the chitin component of PM [51]. The double chitin-binding domains of CnmeGV OFR21 might be more beneficial to virus infection and host liquefaction.

Homologous proteins of ORF21 were also found in 5 alphabaculoviruses. There were DekiNPV, BmNPV, AcMNPV, RaouNPV, and PlxyNPV. A phylogenetic tree was reconstructed based on these conserved domains (Fig 3C). The virus samples were divided into the two major groups of GV and NPV. The *Betabaculovirus* genus of CnmeGV was classified into the NPV group rather than the group of GV, which suggested possible horizontal gene transfer (HGT) occurring in ORF21 of CnmeGV. Two hypotheses are proposed for ORF21 introduction in CnmeGV: 1, the CnmeGV acquired the ORF21 gene from NPV during co-infection of *C*. *medinalis*; and 2, the CnmeGV acquired from the host itself and the viruses might acted as vectors of HGT between insects or animals [52]. Although the transposable elements (TEs) had not been detected in the ORF21 protein, a poly-glutamine residue of trinucleotide repeat ([CAA]_13_) at 156–168 aa was found. This polyglutamine-containing protein appeared to be over-represented in spliceosome components [53].

### Relationships with other baculoviruses

**Fig 5:**
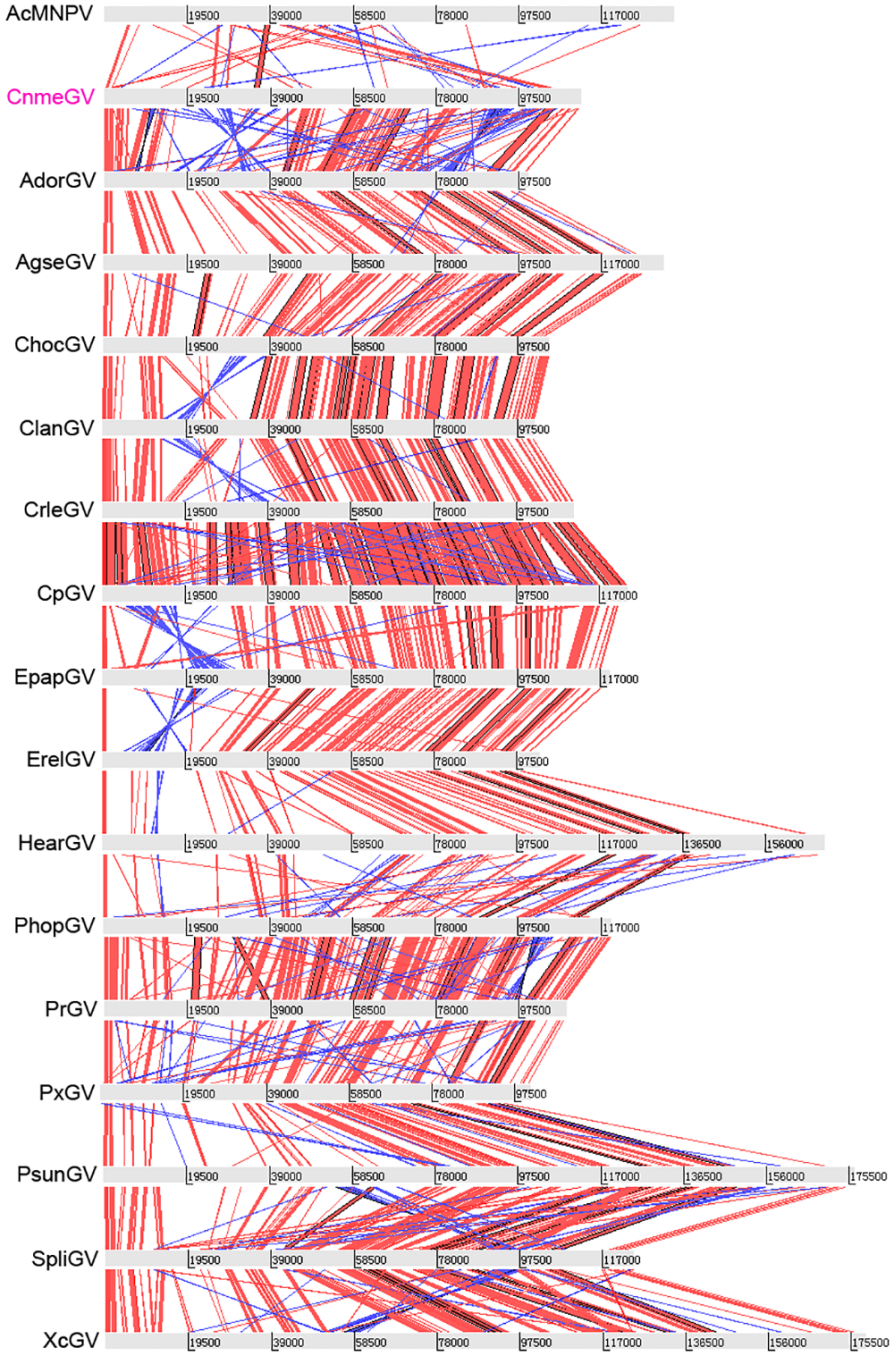
Syntenic map of CnmeGV and other baculovirus genomes. The illustration shows the comparison of gene colinearity based on genome physical positions and protein similarities among 17 GVs and AcMNPV. Each genome is represented by a grey line where nucleotide positions are indicated (kb). Red stripes connecting the genomes indicate syntenic regions in the same strand, whereas blue stripes indicate syntenic regions in opposite strands (inversions). Color intensity is proportional to %identity (darker is more conserved).

Gene colinearity was analyzed by comparing CnmeGV to all the other sequenced GVs and type species of the *Alphabaculovirus* genus, AcMNPV, using Artemis Comparison Tool. Syntenic maps of CnmeGV and other baculovirus genomes were constructed through tBlastX comparison between genomes with blue stripes indicating inversions and colour intensity to reveal the different percentages of identities [20]. The conserved gene colinearity of all 17 GV genomes and the poorly conserved synteny between GVs and AcMNPV are listed in Fig 5. It was apparent that the synteny maps were conserved among betabaculovirus species differing from that of alphabaculoviruses with greater gene order correlation among the CnmeGV and other GVs and some inversions and drifts. Nevertheless, CnmeGV is different from the rest of the GVs by two main gene block inversions about 19.8 kb and 16.8 kb. Inversion of large portions of the genomes was observed in a region between nt 17931–37024 and 81922–98743, that contained eight major ORFs: *p74*, *ubi*, *p106*, *odv-e66*, *alk-exo*, *dna ligase*, *lef-9* and *dnapol*. Most of the differences in the organization among GVs genomes could be explained by insertions and deletions that contributed to the plasticity of the viral population [54, 55].

**Fig 6:**
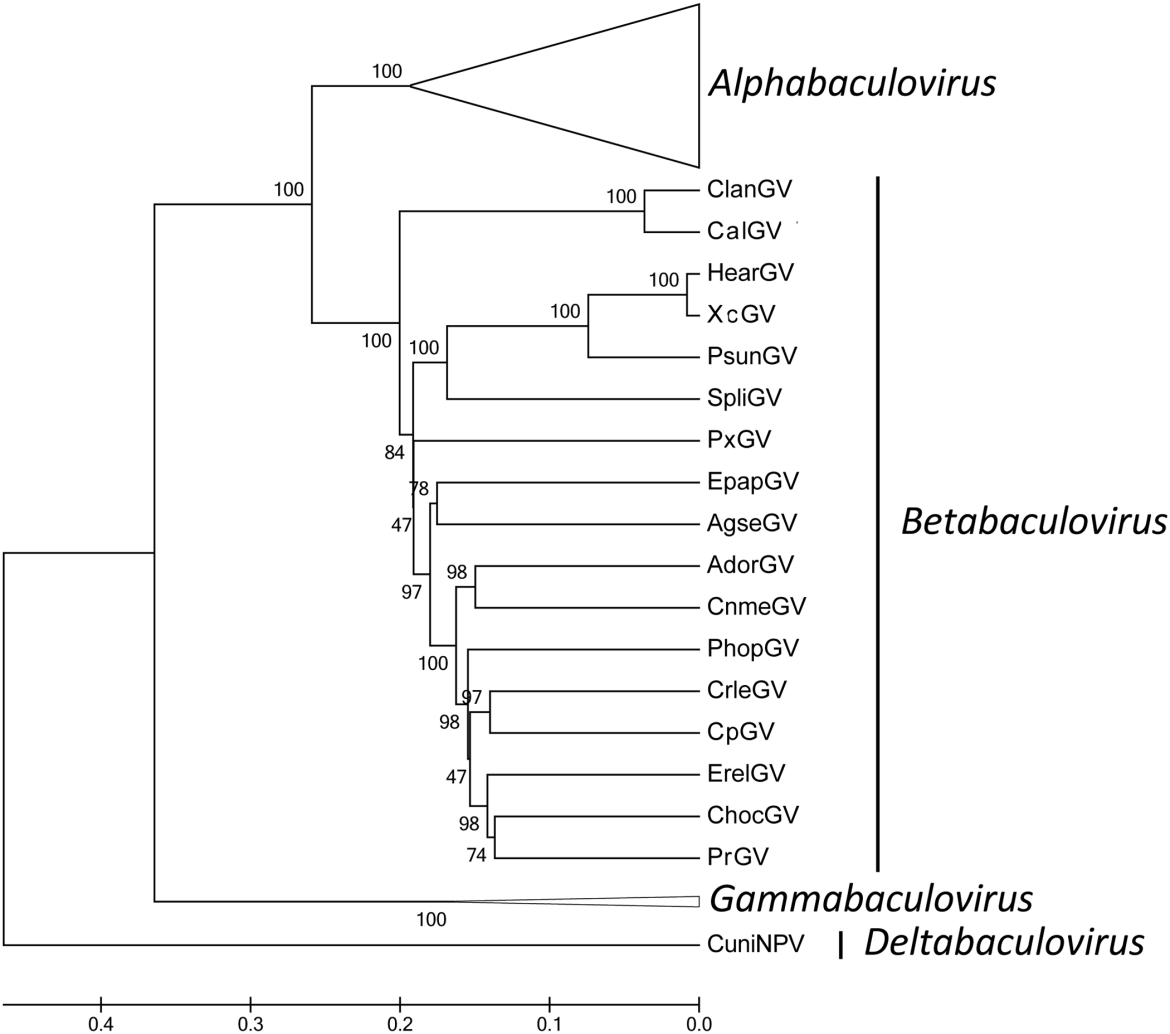
UPMGA tree for all baculovirus. Cladogram based on amino acid sequences of the partial *polh/gran*, *lef-8* and *lef-9* genes in all complete baculovirus genome sequences. We collapsed all the Gammabaculovirus and Alphabaculovirus. The phylogenetic tree was inferred using MEGA 5.1 program.

The neighbor-joining (NJ) and unweighted pair-group method with arithmetic means (UPMGA) trees were generated using the concatenated amino acid sequences of the partial *polh/gran*, *lef-8* and *lef-9* from 68 baculovirus genomes. The UPMGA tree revealed higher bootstrap values (Fig 6 and S1 Fig). The obtained cladogram reproduced the grouping of four genera reflecting the current systematic assignment of the virus family [56]. As expected, CnmeGV was grouped in the *Betabaculovirus* genus. A close relationship between CnmeGV with AdorGV was supported by high bootstrap value. In previous reports betabaculoviruswas mainly divided in two well separated monophyletic clades, Clade “a” and Clade “b”. GVs of Clade “a” were isolated mainly from noctuidae hosts, while those of Clade “b” isolated from other hosts [57]. But the cladogram obtained in this work based on 17 complete GV genomes did not support the division of betabaculovirus in two separated monophyletic clades. The same result was also shown by other authors who constructed the tree using all core genes or *polh/gran*, *lef-8* and *lef-9* genes [58, 17]. Additionally, compared to the evolution and phylogenetic utility in lepidoptera, there are no direct correlation between the classification of insect and host’s virus [59].

## Conclusion

In this study, the first crambidae host-isolated betabaculovirus CnmeGV was sequenced and characterized. Its genome encodes 133 putative ORFs including 37 core genes from baculoviurs. In addition, it contained 32 unique genes that were not shared with the rest of the family with unknown functions. The unique ORF28 protein contained a specialized zinc finger-like domain and a coiled-coil region hypothesized to involve special functions. Fifty one (51) TRs and 6 *hrs* were identified interspersed throughout the genome. ORF21 presented two peritrophin-A domains of CBM_14 that were beneficial to virus infection and host liquefaction. There was also evidence of HGT events from *Alphabaculovirus* to *Betabaculovirus*. Phylogenetic analysis revealed that the CnmeGV is a new *Betabaculovirus* species closely related to AdorGV. The cladogram obtained in this work grouped the 17 complete GV genomes into one monophyletic clade.

## Materials and Methods

### Virus and viral DNA separation

This study was carried out on private land (E: 119.388888, N: 32.479142), the owner permitted us to conduct the study on this site. CnmeGV was isolated from a larva of the rice leaffolder *C*. *medinalis* collected in 2008 and stored in the lab. The virus was not an endangered or protected species. It was multiplied in the laboratory by feeding the second instar larvae with CnmeGV OBs. Infected larvae were homogenized with ddH_2_O, filtered through four layers of gauze, and centrifuged at 7727 x g for 10min. The pellet was suspended in 0.5% (w/v) SDS and centrifugation steps were repeated 5 times until the liquid became clear. Then, the pellet was suspended in 40–65% sucrose gradient and centrifuged at 400 x g for 10 min to remove the debris of larvae tissue. Finally, the OBs were collected in ddH_2_O. Viral DNA was extracted according to Wang et al [60]. Its integrity and identity was analyzed by Nano Drop and Agilent 2100 Bioanalyzer.

### DNA sequencing and analysis

The CnmeGV genomic DNA was sequenced with PacBio RS II at Nextomics inWuhan of China and assembled *de novo* using HGAP2.2.0 [61, 62]. The annotation was performed using RAST [63] to identify the ORF that started with a methionine codon (ATG). The criterion for defining an ORF was the size of at least 150 nt (50 aa) with minimal overlap. Homology searches were done using BlastP in database of NCBI. The complete genome was compared with other betabaculovirus genomes using the Artemis Comparative Tool (ACT) [64] (http://www.sanger.ac.uk/resources/software/act/) and the tBlastX program. The Tandem Repeats Finder [35] (http://tandem.bu.edu/trf/trf.html) was used to locate and analyze tandem repeats. The REPuter program [65] (http://bibiserv.techfak.uni-bielefeld.de/reputer) was applied to analyze homologous repeat regions. The secondary DNA structure and alignment of these sequences were predicted with the DNAMAN 8 and ClustalX programs [66].

The phylogenetic analysis was based on amino acid sequences of 3 core genes (*polh/gran*, *lef-8*, *lef-9*) from CnmeGV and the other 67 baculoviruses listed in NCBI genome database. NJ and UPGMA phylogenetic trees (1000 bootstrap replicates) were inferred from the amino acid sequence alignments by using MEGA, version 5.1.

## Supporting Information

S1 Fig Phylogenetic analysis using the predicted amino acid sequences of the partial *polh/gran*, *lef-8* and *lef-9* genes. The NJ tree is shown. Numbers above or below the nodes are bootstrap values showing the statistical reliability of bootstrapping with 1,000 replicates.
(DOCX)

S1 Table Analysis of putative CnmeGV ORFs with homologous ORFs from databases of NCBI. ^♦^Nucleotide position of putative ORFs and the orientation of transcription are shown in arrows. The gene names are shown in the second column and italicized. The symbols represent the following; *Calculation of amino acid identities (%) in homologous ORFs was based on BlastP. Y ORFs unique to CnmeGV, N ORFs common to other baculoviruses.
(DOCX)
