# Supplementary material for: Genome of Cnaphalocrocis medinalis Granulovirus, the First Crambidae-Infecting Betabaculovirus Isolated from Rice Leaffolder to Sequenced
Source: PLoS One. 2016 Feb 5;11(2):e0147882. doi: 10.1371/journal.pone.0147882 (PMC4746121; doi:10.1371/journal.pone.0147882)
Supplement: S1 Fig — The NJ tree is shown. Numbers above or below the nodes are bootstrap values showing the statistical reliability of bootstrapping with 1,000 replicates. (DOCX) [file pone.0147882.s001.docx]

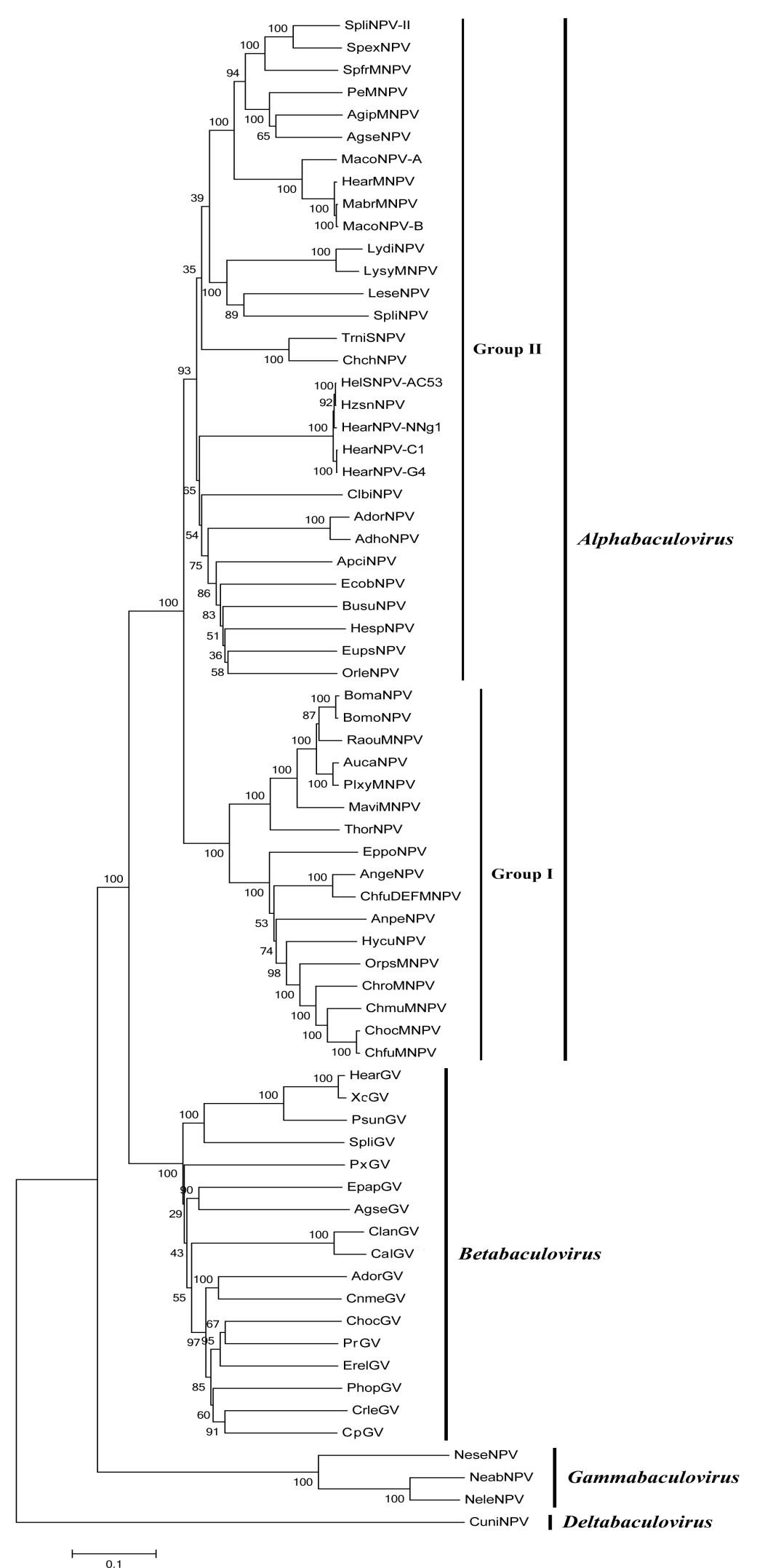


**S1 Fig. Phylogenetic analysis using the predicted amino acid sequences of the partial *polh/gran*, *lef-8* and *lef-9* genes.** The NJ tree is shown. Numbers above or below the nodes are bootstrap values showing the statistical reliability of bootstrapping with 1,000 replicates.
