# Supplementary material for: Genome of Cnaphalocrocis medinalis Granulovirus, the First Crambidae-Infecting Betabaculovirus Isolated from Rice Leaffolder to Sequenced
Source: PLoS One. 2016 Feb 5;11(2):e0147882. doi: 10.1371/journal.pone.0147882 (PMC4746121; doi:10.1371/journal.pone.0147882)
Supplement: S1 Table — ♦Nucleotide position of putative ORFs and the orientation of transcription are shown in arrows. The gene names are shown in the second column and italicized. The symbols represent the following; *Calculation of amino acid identities (%) in homologous ORFs was based on BlastP. Y ORFs unique to CnmeGV, N ORFs common to other baculoviruses. (DOCX) [file pone.0147882.s002.docx]

**S1 Table. Analysis of putative CnmeGV ORFs with homologous ORFs from databases of NCBI** ^♦^Nucleotide position of putative ORFs and the orientation of transcription are shown in arrows. The gene names are shown in the second column and italicized. The symbols represent the following; *Calculation of amino acid identities (%) in homologous ORFs was based on BlastP. Y ORFs unique to CnmeGV, N ORFs common to other baculoviruses.

| **CmneGV ORF number** | **Gene name** | **Nucleotide position (bp)** ^♦^ | **Length aa** | **Homologous gene ID** | **Homology(%)**^*^ | **E-value** | **Unique gene** |
| --- | --- | --- | --- | --- | --- | --- | --- |
| 1 | *granulin* | 1>750 | 249 | AAS86810 | 85 | 6.0e-160 | N |
| 2 | *p78/83* | 747<1217 | 156 | NP_663167 | 30 | 4.0e-11 | N |
| 3 | *pk-1* | 1204>2013 | 269 | YP_003429327 | 58 | 6.0e-113 | N |
| 4 | *CypoGV ORF4* | 2449<3015 | 188 | AIU37214 | 78 | 2.0e-15 | N |
| 5 | *ClanGV_gp005* | 3068>3343 | 91 | YP_004376213 | 48 | 3.0e-13 | N |
| 6 | *ie-1* | 3323<4474 | 383 | YP_003429330 | 46 | 7.0e-80 | N |
| 7 | *PiraGV ORF7* | 4610<4915 | 101 | YP_003429331 | 74 | 1.0e-011 | N |
| 8 | *hypothetical protein* | 4874>5026 | 50 | AGS18773 | 48 | 7.0e-14 | N |
| 9 | *ORF9* | 5799<5998 | 66 |  |  |  | Y |
| 10 | *ORF10* | 6076>6234 | 52 |  |  |  | Y |
| 11 | *ORF11* | 6175<6336 | 53 |  |  |  | Y |
| 12 | *ORF12* | 6426<7076 | 216 |  |  |  | Y |
| 13 | *ORF13* | 7088<7867 | 259 |  |  |  | Y |
| 14 | *ORF14* | 8187<8345 | 52 |  |  |  | Y |
| 15 | *ORF15* | 8531>8785 | 84 |  |  |  | Y |
| *hr1* |  | 9005>9029 |  |  |  |  |  |
| 16 | *odv-e18* | 9049>9330 | 93 | YP_654433 | 51 | 4.0e-08 | N |
| 17 | *p49* | 9331<10719 | 462 | ADO85440 | 60 | 0 | N |
| 18 | *odv-e56* | 10747>11844 | 365 | YP_654435 | 70 | 4.0e-164 | N |
| 19 | *ORF19* | 11845>12063 | 72 |  |  |  | Y |
| 20 | *pep* | 12058<12693 | 211 | YP_654438 | 55 | 2.0e-59 | N |
| 21 | *chit-1* | 12734>13255 | 173 | ACC78173 | 47 | 3.7e-8 | N |
| 22 | *p10* | 13347>14297 | 316 | AJA91657 | 52 | 2.0e-92 | N |
| 23 | *pep-2* | 14333>14797 | 154 | YP_004376244 | 52 | 4.0e-39 | N |
| 24 | *hypothetical protein* | 15700>16983 | 427 | AGS18787 | 30 | 4.0e-5 | N |
| 25 | *lef-2* | 17435>17626 | 63 | NP_891885 | 60 | 6.0e-15 | N |
| 26 | *hypothetical protein* | 17610>17852 | 80 | AEB00316 | 49 | 6.2e-15 | N |
| *hr2* |  | 18006>18763 |  |  |  |  |  |
| 27 | *ORF27* | 18649<18834 | 61 |  |  |  | Y |
| 28 | *ORF28* | 19194>19616 | 140 |  |  |  | Y |
| 29 | *acetyltransferase* | 19693>20292 | 199 | YP_003429378 | 56 | 3.0e-52 | N |
| 30 | *p22.2* | 20468>21010 | 180 | YP_003429376 | 33 | 1.0e-17 | N |
| 31 | *p74* | 22097>24070 | 657 | AIU37268 | 69 | 0 | N |
| *hr3* |  | 24227>24735 |  |  |  |  |  |
| 32 | *sod* | 25199>25699 | 166 | YP_003429374 | 72 | 3.0e-80 | N |
| 33 | *lef-11* | 25731>26054 | 107 | NP_891903 | 60 | 1.0e-30 | N |
| 34 | *39k* | 26017>27180 | 387 | NP_872503 | 31 | 3.0e-12 | N |
| 35 | *CypoGV ORF56* | 27215<27388 | 57 | NP_148840 | 63 | 2.0e-9 | N |
| 36 | *odv-e43* | 27411<28484 | 357 | ADO85472 | 66 | 9.0e-171 | N |
| 37 | *ubi* | 28574>28858 | 94 | YP_654460 | 83 | 7.0e-48 | N |
| 38 | *p106* | 29075>29764 | 229 | ACI43909 | 1 | 5.0e-39 | N |
| 39 | *PiraGV ORF42* | 29774<32290 | 838 | YP_003429366 | 29 | 2.0e-73 | N |
| 40 | *hypothetical protein* | 32318>32518 | 66 | NP_891893 | 45 | 5.0e-8 | N |
| 41 | *ChocGV_gp032* | 32585<33004 | 139 | YP_654453 | 34 | 9.0e-10 | N |
| 42 | *mp-nase* | 33040<34365 | 441 | ADO85463 | 43 | 4.0e-102 | N |
| 43 | *hypothetical protein* | 34431<34754 | 107 | NP_891883 | 58 | 1.0e-36 | N |
| 44 | *odv-e66* | 34799>37066 | 755 | NP_891882 | 66 | 0 | N |
| 45 | *PlxyGV ORF29* | 37067<37228 | 53 | NP_068248 | 57 | 3.0e-15 | N |
| 46 | *pif-3* | 37269<37652 | 127 | YP_003429354 | 44 | 6.0e-29 | N |
| 47 | *ChocGV_gp024* | 37714>38367 | 217 | YP_654445 | 28 | 6.0e-20 | N |
| 48 | *PiraGV ORF27* | 38459<39391 | 310 | YP_003429351 | 35 | 1.0e-12 | N |
| 49 | *F-protein* | 39585<41369 | 594 | AIU36819 | 52 | 0 | N |
| 50 | *ORF50* | 41488<41970 | 160 |  |  |  | Y |
| 51 | *ORF51* | 42251<42562 | 103 |  |  |  | Y |
| 52 | *ORF52* | 42708<43511 | 267 |  |  |  | Y |
| 53 | *ORF53* | 43541<43837 | 98 |  |  |  | Y |
| 54 | *pif-2* | 43933>45075 | 380 | YP_654456 | 65 | 0 | N |
| *hr4* |  | 45100>45374 |  |  |  |  |  |
| 55 | *ORF55* | 45246>45404 | 52 |  |  |  | Y |
| 56 | *ORF56* | 45446<45898 | 150 |  |  |  | Y |
| 57 | *CypoGV ORF62* | 45846<46115 | 89 | NP_148846 | 56 | 9.0e-11 | N |
| 58 | *p47* | 46195>47391 | 398 | AGS18816 | 61 | 3.0e-165 | N |
| 59 | *bv-e31* | 47426>48049 | 207 | YP_003429381 | 77 | 1.0e-96 | N |
| 60 | *p24* | 48153>48604 | 150 | YP_654473 | 65 | 4.0e-40 | N |
| 61 | *38.7kd* | 48669<48980 | 103 | ADO85487 | 42 | 7.0e-12 | N |
| 62 | *lef-1* | 48961<49662 | 233 | YP_654476 | 59 | 8.0e-96 | N |
| 63 | *pif-1* | 49734>51326 | 530 | AGS18821 | 51 | 0 | N |
| 64 | *fgf-1* | 51337<52059 | 240 | ADO85490 | 45 | 2.0e-55 | N |
| *hr5* |  | 52307>52607 |  |  |  |  |  |
| 65 | *bro-a* | 52826<54265 | 479 | YP_002268049 | 76 | 0 | N |
| 66 | *ORF66* | 54587<54910 | 107 |  |  |  | Y |
| 67 | *PiraGV ORF64* | 55166>55552 | 128 | YP_003429388 | 44 | 2.0e-22 | N |
| 68 | *lef-6* | 55571<55894 | 107 | YP_654481 | 42 | 3.0e-11 | N |
| 69 | *dbp* | 55936<56868 | 310 | YP_003429390 | 26 | 2.0e-35 | N |
| 70 | *hypothetical protein* | 56882<57151 | 89 | ADO85495 | 46 | 1e-09 | N |
| 71 | *hypothetical protein* | 57084<57734 | 216 | ADO85496 | 34 | 1.0e-38 | N |
| 72 | *p45/p48* | 57780>58916 | 378 | YP_003429392 | 70 | 0 | N |
| 73 | *p12* | 58990>59364 | 124 | YP_006908587 | 42 | 1.0e-18 | N |
| 74 | *p40* | 59401>60558 | 385 | YP_003429394 | 55 | 8.0e-135 | N |
| 75 | *p6.9* | 60574>60747 | 57 | AGS18831 | 76 | 7e-12 | N |
| 76 | *lef-5* | 60841<61614 | 257 | YP_003429396 | 64 | 2.0e-105 | N |
| 77 | *38k* | 61735>62553 | 272 | YP_003429397 | 63 | 7.0e-114 | N |
| 78 | *odv-e28* | 62513<62851 | 112 | YP_003429398 | 50 | 4.0e-33 | N |
| 79 | *helicase* | 62985>66473 | 1162 | YP_003429399 | 42 | 0 | N |
| 80 | *odv-e25* | 66605<67252 | 215 | NP_891929 | 67 | 7.0e-89 | N |
| 81 | *p18* | 67376<67855 | 159 | YP_003429401 | 47 | 3.0e-39 | N |
| 82 | *p33* | 68200>68718 | 172 | YP_003429402 | 67 | 7.0e-103 | N |
| 83 | *lef-4* | 68860<70308 | 482 | YP_003429404 | 52 | 7.0e-162 | N |
| 84 | *vp39* | 70399>71292 | 297 | YP_003429405 | 55 | 3.0e-97 | N |
| 85 | *odv-e27* | 71370>72272 | 300 | YP_003429406 | 59 | 2.0e-117 | N |
| 86 | *ORF86* | 72304>72555 | 83 |  |  |  | Y |
| 87 | *PsunGV_gp117* | 72644<73195 | 183 | YP_003422456 | 35 | 1.0e-9 | N |
| 88 | *ChocGV_gp078* | 73042<73752 | 236 | YP_654499 | 35 | 4.0e-28 | N |
| 89 | *ORF89* | 73811>74089 | 92 |  |  |  | Y |
| 90 | *vp91* | 74111<75916 | 601 | ADO85514 | 38 | 3.0e-147 | N |
| 91 | *tlp20* | 75888>76385 | 165 | YP_003429410 | 28 | 5.0e-6 | N |
| 92 | *ORF92* | 76402<76794 | 130 |  |  |  | Y |
| 93 | *ORF93* | 76901>77122 | 73 |  |  |  | Y |
| 94 | *bro-b* | 77399>78862 | 487 | YP_009116946 | 69 | 1.0e-162 | N |
| 95 | *PsunGV_gp111* | 79100>79582 | 160 | YP_003422450 | 45 | 3.0e-41 | N |
| 96 | *ChocGV_gp082* | 79800>80417 | 205 | YP_654503 | 62 | 4.0e-84 | N |
| 97 | *gp41* | 80410>81249 | 279 | NP_148888 | 65 | 3.0e-127 | N |
| 98 | *ac78like* | 81272>81586 | 104 | YP_654506 | 55 | 6.0e-10 | N |
| 99 | *vlf-1* | 81573>82676 | 367 | YP_003429414 | 76 | 0 | N |
| 100 | *ChocGV_gp088* | 82693>82947 | 84 | YP_654509 | 62 | 7.0e-20 | N |
| 101 | *ac81* | 83580>84641 | 353 | YP_654527 | 34 | 3.0e-23 | N |
| 102 | *ORF102* | 84685<84891 | 68 |  |  |  | Y |
| 103 | *rr1* | 84890>85291 | 133 | NP_663284 | 31 | 2.0e-9 | N |
| 104 | *alk-exo* | 85352<86551 | 399 | YP_008720057 | 39 | 2.0e-76 | N |
| 105 | *ChocGV_gp103* | 86735<87031 | 98 | YP_654524 | 62 | 6.0e-29 | N |
| 106 | *fgf-2* | 87139>88491 | 450 | AGS18860 | 44 | 1.0e-60 | N |
| 107 | *ORF107* | 88556<88774 | 72 |  |  |  | Y |
| 108 | *ORF108* | 89086<89451 | 121 |  |  |  | Y |
| 109 | *DNA ligase* | 89589>91340 | 583 | ABC67301 | 58 | 0 | N |
| 110 | *fp* | 91483<91938 | 151 | NP_891955 | 47 | 3.0e-36 | N |
| 11 | *lef-9* | 92040<93503 | 487 | YP_003429423 | 72 | 0 | N |
| 112 | *iap-1* | 94128<94979 | 283 | YP_004376308 | 45 | 3.0e-108 | N |
| 113 | *pif-6* | 95033<95830 | 265 | AKS25443 | 61 | 4e-18 | N |
| 114 | *ORF114* | 95898>96443 | 181 |  |  |  | Y |
| 115 | *ORF115* | 96452>96745 | 97 |  |  |  | Y |
| 116 | *ORF116* | 96772>96975 | 67 |  |  |  | Y |
| 117 | *HearGV_gp069* | 96972<97271 | 99 | YP_001649051 | 43 | 1.0e-21 | N |
| 118 | *desmoplakin* | 97366<99099 | 577 | YP_003429418 | 25 | 3.0e-39 | N |
| 119 | *dnapol* | 99254>102511 | 1085 | YP_009091945 | 47 | 0 | N |
| 120 | *ChocGV_gp089* | 102508<102975 | 155 | YP_654510 | 57 | 1.0e-54 | N |
| *hr6* |  | 103152>103368 |  |  |  |  |  |
| 121 | *ORF121* | 103162>103356 | 64 |  |  |  | Y |
| 122 | *lef-8* | 103422<106049 | 875 | YP_003429434 | 63 | 0 | N |
| 123 | *ORF123* | 106210>106602 | 130 |  |  |  | Y |
| 124 | *ORF124* | 106672<106878 | 68 |  |  |  | Y |
| 125 | *ac53* | 106880>107281 | 133 | NP_891968 | 62 | 1.0e-59 | N |
| 126 | *hypothetical protein* | 107265<108041 | 258 | YP_004376324 | 32 | 6.0e-24 | N |
| 127 | *ORF127* | 108098<108253 | 51 |  |  |  | Y |
| 128 | *PiraGV ORF115* | 108280<108474 | 64 | YP_003429439 | 55 | 2.0e-17 | N |
| 129 | *lef-10* | 108449>108682 | 77 | ADO85544 | 61 | 3.0e-18 | N |
| 130 | *vp1054* | 108693>109559 | 288 | YP_003429440 | 58 | 5.0e-116 | N |
| 131 | *ORF131* | 109578>109802 | 74 |  |  |  | Y |
| 132 | *fgf-3* | 109813>110973 | 386 | YP_003429442 | 49 | 2.0e-50 | N |
| 133 | *me-53* | 111092>111927 | 278 | YP_003429444 | 53 | 7.0e-116 | N |
